# Mental suffering, based on the experiences of people who are mentally ill

**DOI:** 10.1017/S1463423625100121

**Published:** 2025-06-25

**Authors:** Carla Aparecida Arena Ventura, Marciana Fernandes Moll, Camila Kaori Hayashi, Bruna Sordi Carrara, Igor de Oliveira Reis

**Affiliations:** 1 Full Professor, Department of Psychiatric Nursing and Human Sciences, Ribeirão Preto School of Nursing, University of São Paulo, São Paulo, Brazil; 2 Director, PAHO/WHO Collaborating Centre for Nursing Research Development-Brazil Coordinator, Unit of the Institute of Advanced Studies, Ribeirão Preto, Brazil; 3 CCHR Fulbright Visiting Scholar - Center for Civil and Human Rights, Notre Dame University, Notre Dame, USA; 4 Inaugural Fellow Leaders for Health Equity Program, George Washington University/Senior Atlantic Fellow for Health Equity, Washington, USA; 5 Professor Doctor, Faculty of Nursing, State University of Campinas, Campinas, Brazil; 6 Nursing student, Faculty of Nursing, State University of Campinas, Campinas, Brazil; 7 Post-doctoral student, Department of Psychiatric Nursing and Human Sciences, Ribeirão Preto School of Nursing, University of São Paulo, São Paulo, Brazil; 8 PhD student, Department of Psychiatric Nursing and Human Sciences, Ribeirão Preto School of Nursing, University of São Paulo, São Paulo, Brazil

**Keywords:** Life story, mental suffering, primary health care

## Abstract

**Objective:**

To understand mental suffering from the point of view of the people affected.

**Method:**

A qualitative study was carried out with 22 users of Primary Health Care units in Ribeirão Preto, São Paulo, Brazil. The data were collected through individual interviews using the Oral Life History technique and analysed using Thematic Analysis.

**Results:**

Two categories emerged: ‘Vulnerabilities in the life history of people with mental suffering’ and ‘Perceiving and living with suffering and/or mental disorder’. The experience was permeated by situations of violence, poverty and abandonment, from childhood to adulthood. The recognition of mental suffering and its consequences was based on behavioural changes and work difficulties, which did not lead them to seek immediate treatment. The difficulty of living with suffering and/or mental disorder is directly related to adherence to treatment.

**Final Considerations:**

Subjective aspects present in human life are still disregarded and the late search for professional help seems to result in the stigma and self-stigma of people with mental suffering and/or disorders.

## Introduction

Mental suffering refers to situations experienced due to stress, trauma, worry, loneliness, bereavement or other emotional challenges and vulnerabilities, such as episodes of violence/abuse, anguish, emotional and psychological pain, experienced uniquely by people and can therefore vary in intensity and duration (Del’Olmo & Cervi, 2017).

It’s important to recognize that mental suffering can be part of the human experience, especially when considering contemporary psychosocial problems – unemployment, economic and political crises, among others (Viapiana; Gomes & Albuquerque, 2018). However, by significantly interfering with daily life and well-being, it can trigger or be associated with a mental disorder. This refers to a clinical diagnosis, being classified into Common Mental Disorders, such as anxiety, depression and Post-Traumatic Stress Disorder; and Severe Mental Disorders, such as psychotic conditions, impulsive-compulsive spectrum and personality disorders (Brasil, 2011). The causes are the result of a combination of factors, including genetic, neurobiological, psychological, environmental, social, cultural, personality, cognitive and emotional development, physical health problems and substance use and abuse (Patel *et al.*, 2016).

Systematic reviews of studies carried out in different countries show that people with mental illness have a poor quality of life, higher rates of illiteracy, comorbidities, low schooling and income, premature mortality and lower life expectancy (Alemu *et al*., 2024; Chan *et al.*, 2023). The experience is permeated by stigma, self-stigma, violence, isolation, difficulty in accessing general and specialized health services, lack of family and social support and suicidal ideation (Ventura *et al.*, 2023; Hernandez Fernandes *et al.*, 2022). In addition, the needs of people suffering from mental illness go beyond biotechnical care, also seeking recognition in society as someone who is biopsychosocially capable of making decisions and enjoying rights (Anjos *et al.*, 2015; Marques *et al.*, 2022).

To minimize harm and promote people’s mental health, the World Health Organization (WHO) and the Pan American Health Organization (PAHO) have developed initiatives focused on professionals and institutions. The WHO, through a Plan of Action, emphasizes the following objectives: strengthening effective leadership and governance for mental health; providing comprehensive, integrated and responsive mental health and social care services in community settings; implementing mental health promotion and prevention strategies; and strengthening information systems, evidence and research in mental health (WHO, 2017). In addition, PAHO advocates the use of professional approaches based on human rights and equity, through reflective, educational and care practices, which involve moments of listening and expression of those involved in therapy and identification of singularities, from the context of the subject’s life and not from a social construct (PAHO, 2023).

It should be emphasized that, in order for these actions to be more effective, the development of studies such as this one is essential because by giving voice and visibility to people with mental suffering and/or disorders, it is possible for them to be seen and feel like active participants in their own care, promoting empowerment and autonomy (Kohrt *et al.*, 2021). In addition, they can ensure that practices aimed at psychosocial rehabilitation are re-signified, based on the needs of the subjects, and thus incorporate evidence aimed at facilitating equity and respect for human rights in the context of these people’s lives. The aim of this study, therefore, was to understand mental suffering from the point of view of the people affected.

## Method

### Study design and location

This research is part of a cross-sectional project that aims to investigate the presence of stigma towards people with mental disorders and substance use problems in PHC in Brazil. This project was implemented in Family Health Units, where there is continuous care aimed at promoting, protecting and recovering health, which is offered by a multi-professional team and has the Family Health Strategy as the protagonist. The quantitative phase was implemented first and losses, during data collection, occurred due to different situations, such as: participants were not found at the health unit because they had not waited for care or had already finished having care; participants did not return to continue data collection, when they were called for their appointment during the interview or when they gave up participating when they realized that their participation did not meet their expectations.

But, this study refers to the exploratory stage of the project, in which qualitative data was collected from Family Health Strategy users at six Family Health Units in the interior of São Paulo, Brazil.

The dissemination of the research in the data collection scenario took place as follows: during the data collection of the quantitative stage, participants were asked about their availability to participate in the qualitative stage and those who were interested, the researchers registered their telephone numbers and, during the qualitative data collection period, the researchers contacted them to arrange an individual interview.

Considering that stigma is a barrier to people seeking and receiving help, the decision was made to include users who had not been formally diagnosed with a mental disorder by health professionals, that is, those in situations of mental suffering. These users contacted the researchers and volunteered to take part in the study after seeing the posters displayed inside the USFs publicising the research.

### Participants

The participants were users of the family health unit over 18 years of age and diagnosed with a mental disorder or dependence on psychoactive substances and users of the family health unit over 18 years of age who do not have a confirmed diagnosis, but who have a score suggestive of a common mental disorder, after answering the Self-Reporting Questionnaire (SRQ-20) (Beusenberg; Orley & World Health Organization, 1994). People using psychoactive substances who were under the acute effect of using licit or illicit drugs or in crisis situations were excluded.

### Data collection

Two researchers were responsible for collecting the data, which took place from January to September 2021, Covid-19 pandemic occurred during this period, when vaccination in Brazil was beginning. Seven interviews were conducted online via Google Meet. After vaccination had progressed and the institutions (University and Municipal Health Department) had authorized a return to academic and research activities, the research team continued to collect data in person and scheduled 11 interviews with participants recruited from their respective Family Health Units.

Among the users who were interested in participating in the research, based on the publicity posters posted in the health units, only four were eligible. Therefore, they were subjected to screening questions, such as *What made you identify with this research?* and *Do you have or have you had any experience that generated mental suffering*? Afterwards, they responded to the SRQ-20 and obtained results suggestive of a common mental disorder.

The open interview was used to collect data, as it allows the free expression of participants and is pertinent to be used in studies with a qualitative approach (Morgado, 2013).

The participants were characterized using a sociodemographic questionnaire (gender, age, race/colour, marital status, family composition, schooling, profession/occupation, income) and a clinical questionnaire (diagnosis, drug therapy and hospitalization). The interview then began with the following triggering question: *Tell us a little about your experience of mental suffering and/or mental disorders*. According to the participants’ discourse, other questions pertinent to the proposed objectives of the research were superimposed. The interviews, both online and face-to-face, lasted an average of 40 minutes. In order to ensure quality control and the accuracy of the content, the interviews were recorded and later transcribed verbatim. The audios were then excluded.

### Data analysis

Four researchers were involved in data analysis, two of which were also dedicated to data collection. To analyse the data, we used Braun & Clarke’s thematic analysis technique, which allows for a better understanding of the discourse when investigating social phenomena, as it favours the synthesis of nuclei that make up communication and the construction of representations, thus allowing for better assimilation of the data in the analysis process. Six steps were applied: (1) familiarization of the data (exhaustive reading of the speeches); (2) generation of codes (systematic coding of relevant and important data); (3) search for themes (grouping of selected codes for transformation into possible themes); (4) continuous review of the themes and identification of the possibility of new themes being synthesized; (5) definition of the themes, relating to the analysis and improvement of the specificities of each theme; (6) production of the final report (self-explanatory interpretation with aggregation of data and empirical categories) (Braun & Clarke, 2006).

## Results

### Characterization of the participants

Of the total number of participants (n = 22), there was a predominance of females (86.4%), aged between 42 and 51 (36%), self-declared white (50%), married (54.5%), and with children (63.6%) – specifically one (22.7%), where the participants lived with them (40.9%) and/or with their spouse (40.9%). Most of the participants had completed secondary school (40.9%), half of whom were employed (50%) and the other half were not (50%), and the main profession/occupation was housewife (27.3%), followed by community health worker (18.2%) and cleaner (18.2%). Most of the participants said they earned between R$1,000.00 and R$3,000.00 (72.7%) and a minority said they received government benefits (18.2%), which were distributed between emergency aid (4.5%), continued benefits (4.5%) and family grants (4.5%).

The majority of participants (81.8%) stated that they had been diagnosed with a mental disorder by a doctor, with a predominance of depression (59.1%), followed by anxiety (50%). It is important to highlight that many of them have more than one psychiatric diagnosis. The four participants who did not have a medical diagnosis of mental illness had an average of 13 on the SRQ-20 score.

The length of time participants had lived with the mental disorder was predominantly between 1 and 5 years (36.4%) and more than 10 years (36.4%). The majority (68.2%) were taking medications, the most used being clonazepam (27.3%), sertraline (22.7%) and fluoxetine (13.6%). It is noteworthy that the majority of participants were not undergoing psychological (72.7%) or psychiatric (90%) treatment. Almost a third said they were supporters of alternative treatments (31.8%), understanding religious practice (18.2%) as such.

Most of the participants had not been admitted to a psychiatric hospital during their lives (72.7%) and the specialized place where they had been admitted was the Psychosocial Care Centre (22.7%). All the participants reported using the USF (100%) and many of them make use of multiple health services in primary and secondary health care.

Most of the participants (72.7%) had not been admitted to a psychiatric hospital during their lives, and the specialized place where they had been admitted was the Psychosocial Care Centre (22.7%). All the participants reported using the USF (100%) and many of them make use of multiple health services in primary and secondary health care.

After analysing the content of the interviews, two thematic categories were identified: *Vulnerabilities in the life history of people with mental suffering* and *Perceiving and living with suffering and/or mental disorder*.

### Vulnerabilities in the life history of people with mental suffering

Financial difficulties have marked the lives of these people from childhood to adulthood. This reality facilitated their early entry into the labour market, and there were reports of child labour, as explained below.

*I had a bit of a troubled childhood, I was very precocious in everything, I had no rules, I started working very early, in the old days we were allowed to work very early, right, so at the age of seven I was already washing the pavements, sweeping the backyard, helping my grandfather, collecting recyclables, we went hungry a lot, and I started working very early, I got a job in a butcher’s shop and I stayed there from the age of eight and a half until I was 21. (Family Health Unit User 8)*

*I had a lot of colleagues, but I married young, I married when I was 20 and my job was, first, when I was 15, I worked with my father, who was blind, a ticket seller, and then I got a permanent job. (Family Health Unit User 7)*

Abandonment, violence and sexual abuse were also part of the participants’ lives.

*I’m from the state of Maranhão and I went to Piauí in search of treatment, because there were no resources in my town. At the time I lived with my mum and my brothers. I had a quiet relationship with them, but with my mother, she’s one of those old people, very strict, so there wasn’t much dialogue. In the past, parents used to correct their children a lot by hitting them, so that was bad. (Family Health Unit User 8)*

*My childhood was linked to my family, right, I have two other sisters, an absent father, I didn’t have that father who was present in my son’s life to play ball, to take him to the football stadium, or that father who took his son to a night out, right? And when I was little, I was sexually abused, part of it was basically between the ages of 8, 7, 10, more or less (Family Health Unit User 4).*

One participant reported attending night-time leisure activities and drinking alcohol at an early age.

*My mum was a bit absent because of work, so I grew up and was very precocious, really. So I had my first binge when I was 13, I started going out at night when I was 12, here in Ribeirão. So when I was 22, I was already tired of clubbing, for example (Family Health Unit User 22).*

It is noteworthy that the violence extended into the marital life of one of the participants, as described in the following account.

*Oh, he bullied me a lot! We fought a lot too. (Family Health Unit User 20)*

In general, the participants’ life history is permeated by the presence of family vulnerability expressed predominantly by a lack of economic resources, emotional abandonment, violence and sexual abuse that occurred in childhood, which undoubtedly predisposes them to mental suffering that can become a chronic mental illness.

Given this reality, it is important to understand what made the participants realize their mental illness and how they live with this condition, described in the next category.

### Perceiving and living with suffering and/or mental disorder

Many participants noticed behavioural changes, expressed by strong physical symptoms (sweating, tachycardia, paresthesia) which were enhanced by feelings of incapacity and fear.

*One day I was having an appointment and I had an anxiety attack, I started sweating, I told the doctor that it would pass, I know how to control it, I just need two minutes and I’ll be fine, then he said if it had started in those days, I said no, I’ve had it since I was a child, only I didn’t know in the past, today I know. (Family Health Unit User 3)*

*It was like this, I worked during the day as normal, it was 2003, I woke up in the middle of the night feeling ill, then they rushed me to hospital thinking I was having a heart attack, when I got there the doctor said ‘oh, you could be having a heart attack, at this age you’re having a lot of heart attacks’, but no. It was my pressure fluctuating, my hands freezing, my nose and mouth tingling, all my extremities, that feeling of fainting, my pressure was fluctuating, my hands were freezing, the tips of my nose, my mouth, my hands, everything was tingling, all my extremities, that feeling of discomfort, it felt like I was going to come and go, the feeling of fainting, that I was going to lose consciousness, and from then on my suffering began, it was a great suffering. (Family Health Unit User 10)*

*A little over a year ago, when I went to hand in my CV, I kept looking for a job, wanting an answer and getting nothing. Then my heart would race, I’d want to cry, I’d just want to lie down. (Family Health Unit User 14)*

Changes in sleep, appetite and in the performance of their work duties led the participants to recognize that there was some mental impairment.

*I realised this because I used to get very anxious when I was alone, it would consume me, I’d get distressed, anxious, sometimes I wouldn’t even leave my seat or I’d start inventing things, inventing things to try to use up that energy that was inside me, that I couldn’t control. (Family Health Unit User 1)*

*I didn’t sleep any more, it was sleepless nights, I didn’t eat, I didn’t drink, so it was difficult, even to work, I couldn’t work any more either, then I started to be afraid to go out on the street, then the other part of the panic started, fear of going out on the street and something happening. I didn’t do anything anymore, I didn’t feel like doing anything else, I didn’t feel like doing anything else, my thing was to get home, go into my room and stay quiet. (Family Health Unit User 6)*

In addition, two participants reported that they didn’t realize their mental impairment on their own and that it was their family members who helped them to realize it.

*Because I’d go to sleep and I’d hear voices, I’d be terrified to no end, sometimes I’d hear my mum’s voice change, my heart would race. Because of these things, I knew I wasn’t well. But in truth, I didn’t even ask her for help, I ended up taking medication, thinking it would solve the problem. That’s when she realised that I wasn’t well either. (Family Health Unit User 8)*

*Look, it was a really funny moment, because it was when I went camping in front of a guy’s house in São Paulo. Because I was hearing voices and these voices were telling me that he was cheating on me or something and I went camping in front of his house. Actually, I didn’t even have this click. It was normal for me. It was my family who had the click. Who then hospitalised me. (Family Health Unit User 22)*

The association between initial perceptions of commitment and the birth of children was reported by two participants and another three related it to the death of loved ones.

*Because I was 16, at that time I was getting used to life as a mum, everything. There were times when I got angry with him, when I didn’t want to look after him, or bathe him, or give him a bath. Then I realised that I was in agony with him. Even more so because I worked. (Family Health Unit User 11)*

*I had a pregnancy and I lost the pregnancy and started to have this anxiety. Then I got pregnant with the other one and it gave me a lot of anxiety. After I had him I was afraid to go out with him, I was afraid of someone taking him away from me, you know? Being taken from me, I don’t know, I thought these things. (Family Health Unit User 9)*

*When I lost my father, after a few months, I realised I wasn’t well, I just cried, I didn’t sleep, so I went to see a doctor. (Family Health Unit User 15)*

*It was the moment I lost my father and I felt alone, so I didn’t feel like going out anymore and I stayed in my room for three months and didn’t go out for anything, I didn’t do anything. (Family Health Unit User 18)*

*My son was chemically dependent, addicted, you know, and then it was just suffering. I tried very hard to get him off drugs and I couldn’t, and in the end that’s what killed my son. I still suffer from that to this day, missing my son. Then, when my other son continued with drugs and addiction, I’m just as worried that the same thing will happen as before, so I suffer from this. (Family Health Unit User 13)*

Conflicts and/or breakdowns in affective relationships were also triggers for recognizing psychological problems, as the participants reported below.

*When my little boy was born, I discovered that my husband had been cheating on me. I thought it was my son’s fault that his father had cheated on him, so I put all my anger on the child. I tried to kill him, I tried to kill myself and that’s when I discovered that I had post-natal depression, and that’s when the treatment started. (Family Health Unit User 2)*

*I had sexual desires for other boys, but I kept it to myself. And then, around the age of 18 to 20, I ended up getting into a relationship with a guy, and then, let’s put it this way, I came out to myself. It was love at first sight, but then it didn’t work out, and I went into, I guess I could say, a bit of a depression. (Family Health Unit User 4)*

Although the participants were aware of their condition, it was only when it worsened that they sought help, and their justification for this centred on the lack of resources and tools to deal with it on their own.

*Nowadays, it’s at a slightly more delicate moment, because I had a relapse after the end of my last relationship, but I went back to therapy, I’m taking medication, so it’s stable, but I know that it’s still at that moment when I have to keep my head on straight so that I don’t relapse again. But I can cope better than I did the first time. As today is already a second, stronger crisis, I cope better now than before. (Family Health Unit User 5)*

*It only got worse, until I got a friend who said ‘no, this isn’t right, we’re going to see a psychologist’, and I started psychological treatment, with a psychologist first. (Family Health Unit User 6)*

Generally speaking, realizing that there was a psychological impairment didn’t seem to be enough for people to seek help and this made them associate the changes they identified as a response to events that would occur in their lives (motherhood, bereavement, marital problems or separations, among others), which expresses the presence of self-stigma when it comes to recognizing the psychological impairment and seeking professional help. This condition was confirmed when they reported difficulties living with the mental illness.

*I find it unpleasant when you have an adverse situation that you need to balance, I believe even with my disorder, I have it, but it generates something else that makes you worry. It’s all very stressful. It’s not nice. (Family Health Unit User 1)*

*It’s difficult, because every day, basically, when I put my head on the bed, I think about a lot, a lot of things, things I haven’t done, things I have done, or things I’d like to do. I think I’ve been losing focus a bit, I lose sleep. I do, but sometimes at the insistence of my partner, but if it were up to me, I’d get to the weekend without wanting to get out of bed. (Family Health Unit User 4)*

*The word is painful, I don’t know the right word. Anxiety is something that puts you in a bad mood, you don’t feel like doing anything. (Family Health Unit User 12)*

What’s more, the fear of hearing voices, having panic attacks and feeling unwell seems to make it difficult for participants to live in harmony with themselves and even to look for work.

*I’d like to get a job, right? But I can’t. (Family Health Unit User 20)*

*I get scared, you know? Of the worst happening in my life, because I love my children very much and I’m afraid. (Family Health Unit User 13)*

*It scares me. I live far away from my parents, my family is just me, my husband and my son. Then I’m afraid of losing my father, my mother and not seeing them again, so I’m scared. (Family Health Unit User 9)*

*First it started with depression and panic disorder, then they treated it, but I got worse and worse and worse. They treated one thing and it didn’t resolve it because I saw voices, shadows, you know? When I’m very stressed, the voices are horrible, as well as the panic, the things come, the voices come. I’ve had eight suicide attempts, I almost died, it was by my arms, apart from the other things I’ve taken, poison, these things. Why? I wanted to get out of that pain, that discomfort I felt, just by talking my eyes filled with water, that thing. (Family Health Unit User 10)*

On the other hand, there were participants who did not consider the mental illness to be a hindrance to their lives, which they attributed to the use of alternative therapies, family support, the use of medication and psychiatric treatment.

*Nowadays it’s more peaceful, because of the alternative medicines I use and my mum’s support. (Family Health Unit User 14)*

*Today I live in peace. I had a crisis in May last year, a schizophrenic crisis, but it didn’t require hospitalisation. I’m working, I’m leading my life, I take my medication and I lead my life peacefully, I have nothing to complain about. As for the illnesses, they’re there, they’re being treated and that’s it. (Family Health Unit User 22)*

There was a predominance of participants who reported that they did not live well with their mental illness and those who said they lived well with the condition attributed this to the fact that they were involved in different forms of treatment.

## Discussion

The search for health services, especially those belonging to Primary Health Care (PHC), is more frequent among women and this may justify their predominance as participants in this investigation. In addition, women are more likely to experience mental suffering and develop disorders when compared to men, and this occurs on a global and national scale (Anjos *et al.*, 2015; Smolen & Araújo, 2017; Gutiérrez-Rojas *et al.,* 2020; Quadros, 2020). For women, socioeconomic variables have a greater influence on the development of mental suffering and/or disorders, while for men, the fact that they live alone and are not employed has a greater influence (Quadros, 2020), which does not corroborate the findings of this investigation, in which the unemployment rate among the participants was equal and more than half of the participants have children and live with family members (children, wife or husband).

As for race, half of the participants declared themselves to be white, which is not in line with the findings of scientific investigations that have shown a higher prevalence of black or brown people with mental suffering and/or disorders (Smolen & Araújo, 2017; Gutiérrez-Rojas *et al.,* 2020; Quadros, 2020). There was a predominance of 42- to 51-year-olds (36 per cent) among the participants in this investigation, which is related to the period of middle adulthood that extends from 40 to 65 years of age (Papalia & Feldman, 2013), which does not corroborate the findings of studies that have shown an age range starting at 18 years of age and extending to 39 years of age (Gutiérrez-Rojas *et al.,* 2020; Carpena *et al.,* 2019). This reality expresses the need to pay attention to mental health in adulthood.

The predominance of participants with complete high school education (40.9%) and income between R$1000.00 and R$3000.00 is occurred in this investigation and this does not corroborate the investigations that point out that lower schooling and lower income are related to a higher incidence of mental disorders (Smolen & Araújo, 2017; Quadros, 2020). However, there are important inequalities in Brazil and this research was carried out in the state of São Paulo, which is located in the southeast region, considered developed when compared to other Brazilian regions.

Diagnoses of depression and anxiety predominated among the participants and this was also reported in scientific research that addressed this reality in the Brazilian scenario (Anjos *et al.*, 2015) and worldwide (Gutiérrez-Rojas *et al.,* 2020). The presence of more than one diagnosis of mental disorder was present in this study and is also pointed out in other scientific investigations (Gutiérrez-Rojas *et al.,* 2020; Carpena *et al.,* 2019; Quadros, 2020). The fact that just over half of the participants used drug therapy but were not receiving psychological or psychiatric care seems to be a reflection of the lack of information due to not consulting a professional and not being aware of their real condition (Owie *et al.,* 2018), which seems to be related to personal stigma. On the other hand, adherence to alternative treatment practices, with an emphasis on religious practice, can be beneficial as they help patients to accept their condition and seek improvement (Domingues *et al.*, 2020).

The oral history of life as a collection technique allowed for a more holistic and inclusive view of the participants’ past events (Santos & Silva, 2022). Their stories were marked by situations that predispose to reduced quality of life, such as financial difficulties and early entry into the labour market, which is pointed out in national scientific circles as a driver of mental illness (Assunção; Lima & Guimarães, 2017; Barros *et al*., 2019). In this scenario, it is necessary to consider that environmental and human resources alter the development of human being and can make mental health vulnerable (Sui, Ettema & Helbich, 2022), as was portrayed in this investigation.

Violence, in its various forms, has been present since the childhood of many of the participants studied. This phenomenon is a significant source of mental suffering for those involved – victims, witnesses and even perpetrators, with deep and lasting impacts. In addition to direct participation, repeated exposure to violence can desensitize and lead to the normalization of these behaviours, contributing to a cycle of continuous and invisible violence (Costa; Baptista & Cunha, 2022). Naturalization was perceived in the interviewees’ statements when they reported violence as a form of correction within the family.

In this scenario, sexual violence, experienced in childhood and perpetuated in the adult lives of some participants, deserves attention. These events are often present in the lives of people in situations of vulnerability and concomitant with other forms of violence (Burgić Radmanović, 2020) – such as pregnancy, which, as a result of rape, in addition to sexual abuse, can also have physical and psychological repercussions. However, the order of occurrence cannot be determined, nor can a causal relationship be established (Gusmão *et al.*, 2018). The important thing is to understand these events and consequences, as they can signal the greater vulnerability to mental suffering that victims of sexual abuse are exposed to.

European studies that have explored dysfunctional family relationships and mental illness highlight the relocation of children and adolescents due to abandonment by other family members or even strangers as one of the causes (Zagefka *et al.,* 2021; Tan *et al.,* 2022), culminating in depressive symptoms and psychotic episodes. These situations were also exposed in the history of the participants in this study, as well as other vulnerabilities, such as unemployment and poverty, in which the literature points to a strong relationship with mental suffering and CMD (Kromydas *et al.*, 2021). It should also be noted that men tend to be more affected by transitions or job losses, while poverty can have more significant repercussions for women and people with less education (Kromydas *et al.*, 2021). This can be explained by the socially imposed duties of the genders which, when not achieved, cause damage to mental health (Tankersley *et al.*, 2021).

Even in the face of conditions that have made the participants vulnerable and generated mental suffering throughout their lives, none of them said they had sought professional help, or that family members or people close to them (teachers and others) had taken them for some kind of care. This suggests that these participants may not have believed in or valued mental suffering when they were children. It was only in adulthood, when mental suffering had already set in, with the manifestation of physical symptoms, altered sleep, appetite, behaviour and work performance, that the participants and their families realized their condition was negatively interfering with their quality of life, corroborating other evidence (Barros *et al*., 2019; Assunção; Lima & Guimarães, 2017).

There are still many barriers that prevent people from recognizing suffering and/or mental disorders, one of which is stigma and self-stigma (Hernandez Fernandes *et al.*, 2022). These lead to feelings of guilt, anxiety, anger, disapproval, hopelessness and low self-efficacy, which predispose to greater suffering among people with mental disabilities (Flores, 2020; Koljack; Garcia & Davis, 2020). This also interferes with the provision of care, as Primary Health Care professionals in Brazil still have stigmatizing and negative views towards people with psychotic episodes or who use alcohol and other psychoactive substances (Bobbili *et al.*, 2022), although there are efforts to reduce this problem (Carneiro *et al.*, 2022).

Many participants justified not seeking help due to the lack of resources and tools to deal with this condition on their own, which expresses the importance of the support network that can act both to protect the person and to predispose them to illness since it was mentioned in this investigation that conflicts or the breakdown of affective relationships made some participants realize that they were ill. Valuing the support network in the care process on the part of professionals is therefore essential, as it represents a mitigating factor against mental illness (Gaino *et al.,* 2019; Feng & Astell-Burt, 2016).

The birth of children was also an event that made some participants realize their condition and this is indeed considered a risk factor for changes in mental health (Gaino *et al.,* 2019; Feng and Astell-Burt, 2016), especially among women who have experienced situations that have generated intense emotional suffering throughout their lives.

Even if they realized it, living with mental suffering was difficult and this made it difficult to be in harmony with oneself and even to look for work, as well as favouring low adherence to therapies by many of the participants in this investigation. It should be emphasized that adherence to therapeutic practices is higher among people who don’t consider their mental illness to be a hindrance (Amanai *et al.*, 2022), which corroborates the reports of some participants who say that taking part in different forms of treatment is a strategy for living better with this condition.

By giving the participants a space to speak, it was possible to value them and make them feel important and protagonists of their own stories, or even to be able to see positive versions of themselves (Santos & Silva, 2022). Despite the delicacy and complexity of the subject, the authors of this study strongly believe and rely on other evidence (Branco, 2020; Ribeiro, 2021) to say that the Oral Life History technique allowed the participants to be inserted into a social context that they sometimes don’t feel part of.

As a limitation, the study was carried out in only six Family Health Units in a municipality in the state of São Paulo, and the results cannot be generalized.

## Final considerations

The results of this study show a life history permeated by economic, emotional and family vulnerabilities that generate mental suffering from childhood onwards and, for the most part, culminate in mental disorders. However, this suffering was not considered by the participants, nor by their carers, and the perception of mental distress only occurred when emotional symptoms and damage to work activities set in.

This signals that the subjective aspects of human life are still disregarded, which seems to result in stigma. The search for professional help was also late and this can reflect on self-stigma, generating difficulties in living with suffering and/or mental disorder and in adhering to treatment.

The findings of this study express the need to invest in interventions to work on the stigma that exists in society, through educational and awareness-raising actions, since accentuated psychological problems can be avoided or minimized if the person receives early multidisciplinary follow-up.
